# Retraction Note: MALAT1 regulates the transcriptional and translational levels of proto-oncogene RUNX2 in colorectal cancer metastasis

**DOI:** 10.1038/s41419-025-07490-7

**Published:** 2025-03-05

**Authors:** Qing Ji, Guoxiang Cai, Xuan Liu, Yi Zhang, Yan Wang, Lihong Zhou, Hua Sui, Qi Li

**Affiliations:** 1 Department of Medical Oncology, Shuguang Hospital, Shanghai University of Traditional Chinese Medicine, 201203 Shanghai, China; 2 Department of Colorectal Surgery, Fudan University Shanghai Cancer Center, 200032 Shanghai, China

Retraction Note to: *Cell Death and Disease* 10.1038/s41419-019-1598-x, published online 16 May 2019

The Editors-in-Chief have retracted this article. After the publication of this article, concerns regarding published images were raised. The authors were able to provide explanations and raw data, however, an investigation by the Publisher found a discrepancy in Figure 5e between published and raw data, similarities between Figure 7a (LoVo, shRNA-NC) and 7a (SW260, pcDNA3.1-RUNX2), and similarity between FIgure 5f (PTBP2) and Figure 5d (Snail) of [1].

The Editors-in-Chief therefore have lost confidence in the content presented in this article.

Author, Qing Ji disagrees with this retraction. None of the remaining authors have responded to the Publisher/Editor regarding this retraction.
